# Reframing pediatric hypertension: early life clues and clinical imperatives

**DOI:** 10.1038/s41390-025-04674-x

**Published:** 2025-12-03

**Authors:** Akash Deep, Emma C. Alexander

**Affiliations:** 1 Paediatric Intensive Care Unit, King’s College Hospital, London, UK; 2 Department of Women and Children’s Health, School of Life Course Sciences, King’s College London, London, UK; 3 Paediatric Critical Care Research Group, Department of Surgery and Cancer, Imperial College London, London, UK

Pediatric intensivists often encounter children in the ICU with elevated blood pressure. For some, this is a complication associated with acute illness. Others present on a background of pre-existing hypertension that had gone unnoticed, and importantly, others still may present with hypertension having been admitted to the ICU years back with renal complications, but who did not receive any long-term follow-up.^1^ This pattern challenges our long-standing assumptions about when and how cardiovascular risk begins.

The newly published longitudinal study by Butts-Jackson et al. tracking blood pressure (BP) trajectories in early childhood, contributes meaningfully to this evolving conversation.^2^ This study uses data from 1040 participants in a prospective birth cohort study conducted in North Caroline, United States, between 2005 and 2011. Blood pressure measurements were obtained from patients’ medical records, and demographic variables from questionnaires. The authors identified blood pressure percentile trajectories using group-based trajectory modelling, defining four distinct systolic blood pressure percentile trajectories (lower stable, lower increasing, higher stable, and higher decreasing) and three diastolic percentile trajectories (lower increasing, higher decreasing, and higher stable), as well as describing key variables associated with group assignment.

In particular, this study adds value through its tracking of children’s blood pressure from such a young age, younger than prior studies examining trajectories from older ages (beginning 6–12 years).^3,4^ The study suggests that BP trajectories are already diverging from the age of three, influenced by both biological and social determinants. This finding has profound implications for how we understand and act on early cardiovascular risk.

## Blood Pressure Trajectories Begin Early, But Unequally

The study described distinct BP trajectory patterns in children aged 3–9, shaped not only by perinatal factors like gestational diabetes and pre-pregnancy BMI, but also by variables such as parental education and relationship status. What is striking is the interplay between these domains, biology and social context, working in tandem to shape cardiovascular futures. These associations paint a picture of systemic vulnerability in the face of environmental and social stressors. This finding supports prior work showing that exposure to adverse childhood experiences can influence blood pressure levels in young adulthood.^3^

## Birthing Parent Race and Ethnicity

The study found that birthing parent race and ethnicity were both associated with systolic and diastolic percentile trajectory group assignment. Such disparities may reflect the impact of structural inequities, differential access to preventive care and disparate clinical management. In accordance with the sex differences in blood pressure also described in this study, these results are in concordance with previous work describing increased risk of hypertension in childhood among Black and Hispanic boys.^5^ Other research has shown that socioeconomic status accounts for a significant proportion of observed racial disparities in early life blood pressure trajectories.^6^ It is an imperative for healthcare professionals to consider and seek to address structural healthcare disparities and ensure equal accessibility of services for preventative care especially given the long-term cardiovascular risks conferred by early-life hypertension.^7^

## Monitoring Trajectories and Making Contacts Count

Traditional BP screening often hinges on thresholds and one-off measurements. But the study’s trajectory-based analysis shows that children whose BP trends upward, even within the ‘normal’ range, might already be on a path to later disease. This demands a shift from static cut-offs to dynamic trends. In the ICU, we already monitor trends intensively; why not extend this philosophy to assessment of blood pressure in the paediatric population? Consideration could be given to assigning diagnostic codes in line with BP trajectory groups even in children who do not meet formal criteria for hypertension. NHS England in the UK currently promote the ‘Making Every Contact Count’ initiative, whereby each healthcare staff interaction should ideally also be used to support individual behavior change to reduce disease risk, through brief discussion on health or wellbeing factors.^8^ Acknowledgement and discussion of blood pressure trajectories during clinical contacts would fit well within this philosophy, with potential long-term benefits.

## Real-World Implications

The implications of this study could certainly have clinical relevance. We recall a case of an 11-year-old boy admitted with hypertensive encephalopathy. Post-stabilisation, his records revealed that elevated BP had been intermittently noted for years but never acted upon, as it remained “just below the 95th percentile.” Had trajectory thinking been applied earlier, his outcome might have been different.

The study under discussion offers a framework that aligns with what many clinicians intuitively sense but lack the data to defend: that early deviations, however small, can matter. Another important observation in the real world is children who develop acute kidney injury (AKI) whilst being admitted to PICU as a part of multi-organ failure (sepsis, liver disease, cardiac disease) are rarely followed up for long term sequalae like chronic kidney disease and hypertension, This study reinforces the need to long term follow up of children who develop AKI in the early years.

## Study Limitations

The study is not without limitations, or unanswered questions. The study replies on blood pressure measurements extracted from medical records, which may vary depending on quality of record-keeping. It is also not possible to determine whether these measurements were taken when the child was well, or unwell. Authors prioritized measurements taken close to the time of the child’s birthday, but other approaches or averages could have been used. As acknowledged by the authors, selection bias is present, in particular exclusion of non-English speaking individuals who may have higher risk. In particular, the next step would be associating these trajectory groups with cardiometabolic outcomes.

## What This Means for Research and Policy

Future work must explore whether blood pressure trajectory groups predict hard outcomes including organ damage, cardiovascular events, or even mortality. We also need better integration of genetic, epigenetic, and environmental exposures to unpack causal mechanisms. In particular, children admitted to hospital who experience acute kidney injury should be followed up, especially if there is evidence they may already belong to a high risk-trajectory group. Children who are hospitalised out of their local area may be at increased risk of loss to follow-up. For policymakers, the findings of this study call for a broader lens on child health: one that includes an assessment of maternal health, sociodemographic equity, and prioritises early preventive care.

## Conclusion

If we are to build a healthcare model that is preventive, equitable, and data-informed, studies like this must inform both practice and policy. This study reminds us that elevated BP in a child is rarely an isolated event; is often a reflection of upstream risks we have yet to mitigate. In paediatrics, it is time we view cardiovascular risk not as an event to be managed, but as a trajectory to be altered, starting as early as possible.

## Data Availability

All data generated or analysed during this study are included in this published article.
